# Latent distant metastasis of renal cell carcinoma to skin: A case report

**DOI:** 10.1002/ccr3.2844

**Published:** 2020-04-13

**Authors:** Pretty Singh, Kavita Somani

**Affiliations:** ^1^ Department of Pathology Apollomedics Super Speciality Hospital Lucknow India

**Keywords:** cutaneous metastasis, disease‐free survival, history, latent metastasis, Renal cell carcinoma

## Abstract

Renal cell carcinoma (RCC) is known to recur decades after nephrectomy; however, isolated cutaneous metastasis is rare. Previous clinical history plays vital role. Newly occurring skin lesions in follow‐through patients of RCC should be carefully evaluated, and possibility of its metastasis should be acknowledged. Disease‐free survival postmetastasectomy is possible.

## Graphical abstract

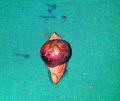

## 1 INTRODUCTION

Renal cell carcinoma (RCC) is a common neoplasm of kidney. The most common sites of metastasis are lung, liver, lymph node contralateral kidney, or adrenal glands. Skin metastases of renal cell carcinoma are very rarely seen (1.0%‐3.3%), and they usually occur as a late manifestation of the disease.^1^ It is known for latent distant metastasis, even after decades of nephrectomy. It may metastasize latently to rarer locations like skin.

We present a rare and unsuspected case of latent cutaneous metastasis of RCC, which occurred a decade postnephrectomy.

## 2 CASE PRESENTATION

A 65‐year‐old male patient presented to the plastic surgery outpatient department of our hospital with a progressively enlarging discolored mole‐like growth on the left frontal region, near the eyebrow, since three months. The gentleman was cosmetically concerned and wanted its excision because of its growing size and recurrent bleed due to friction. He did not have any other ailment and did not give any significant clinical history. In suspicion of a skin neoplasm, likely to be a vascular tumor, the lesion was excised with secure margins and sent to us for histopathological examination.

**Figure 1:**
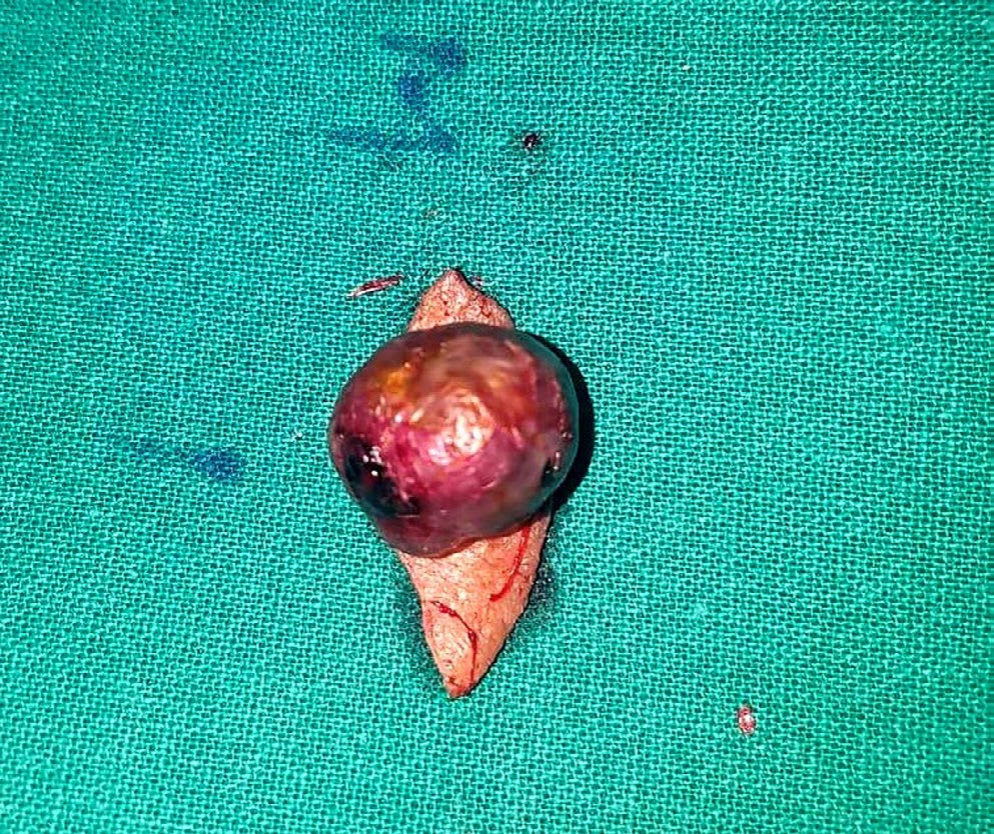
Macroscopic picture of unfixed excised left frontal skin lesion specimen

**Figure 2:**
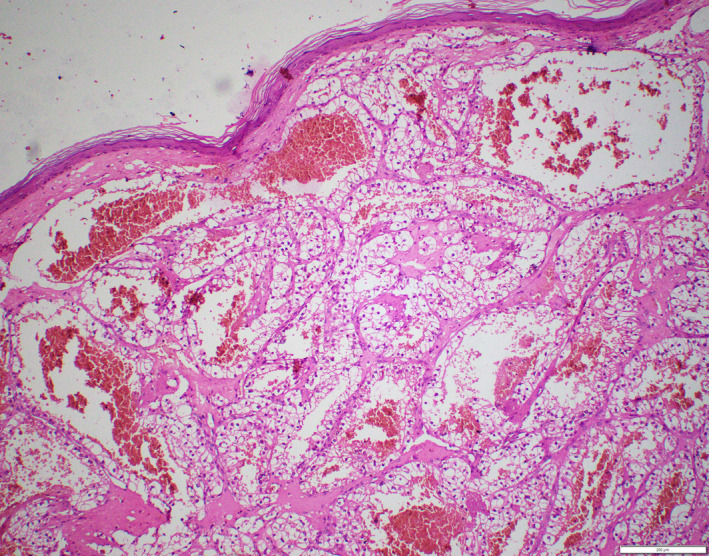
(200×): H&E: Subepithelial lobules of tumor cells with intervening areas of hemorrhage

**Figure 3:**
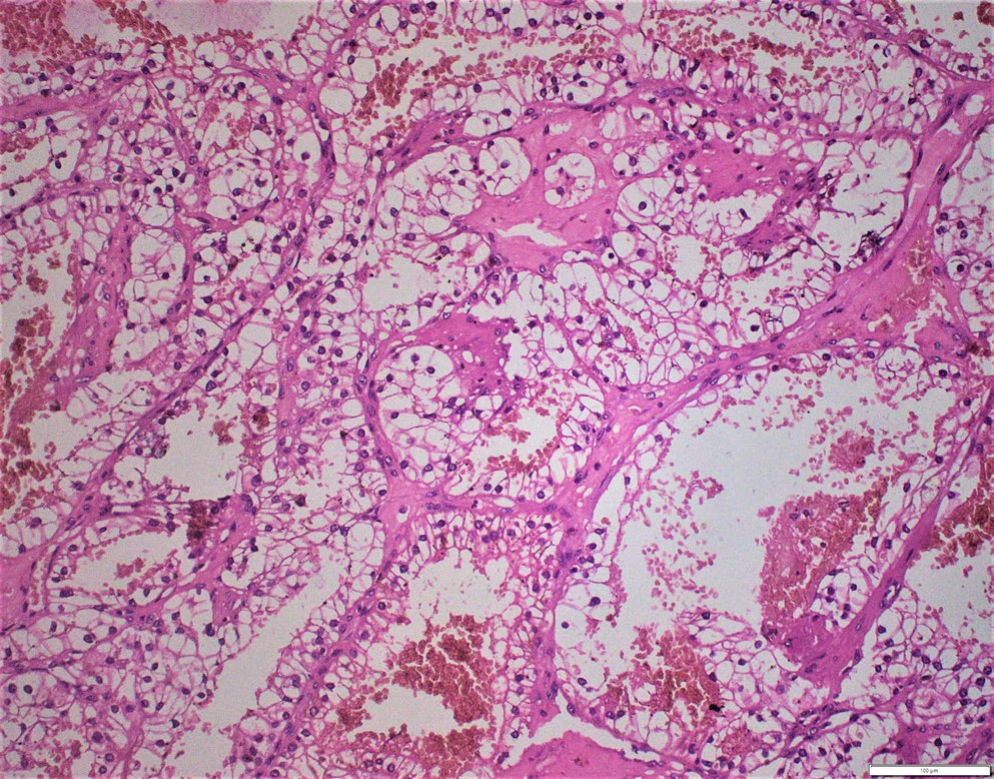
(400×): H&E: Tumor cells with clear cytoplasm and small nuclei and inconspicuous nucleoli

The specimen was received as a grayish brown colored nodular growth over the skin, which measured 1.5 × 1.2 × 1.2 cm (Figure 1). Cut section showed solid gray brown areas with multiple areas of hemorrhage. On histopathological examination, a subepithelial neoplasm was identified that was disposed into nests and lobules with intervening areas of hemorrhage. The tumor cells had round to oval nuclei, with inconspicuous nucleoli and moderate amount of clear cytoplasm. (Figures 2 and 3). All the margins were clear of tumor deposits. The histomorphology of this tumor had a striking similitude with renal cell carcinoma‐clear cell variant. In consideration of this unexpected morphology, we asked the patent if he had any prior history of malignancy that was overlooked in the initial interview. He revealed that 10 years ago he had a clear cell renal cell carcinoma treated with nephrectomy. He was under follow‐up at some other institute with a yearly computed tomographic scan, which had revealed no evidence of recurrence, two months ago. However, we went ahead with immunohistochemistry of the tumor and referred the patient for PET scan for further confirmation to rule out recurrence and other metastatic lesions.

**Figure 4:**
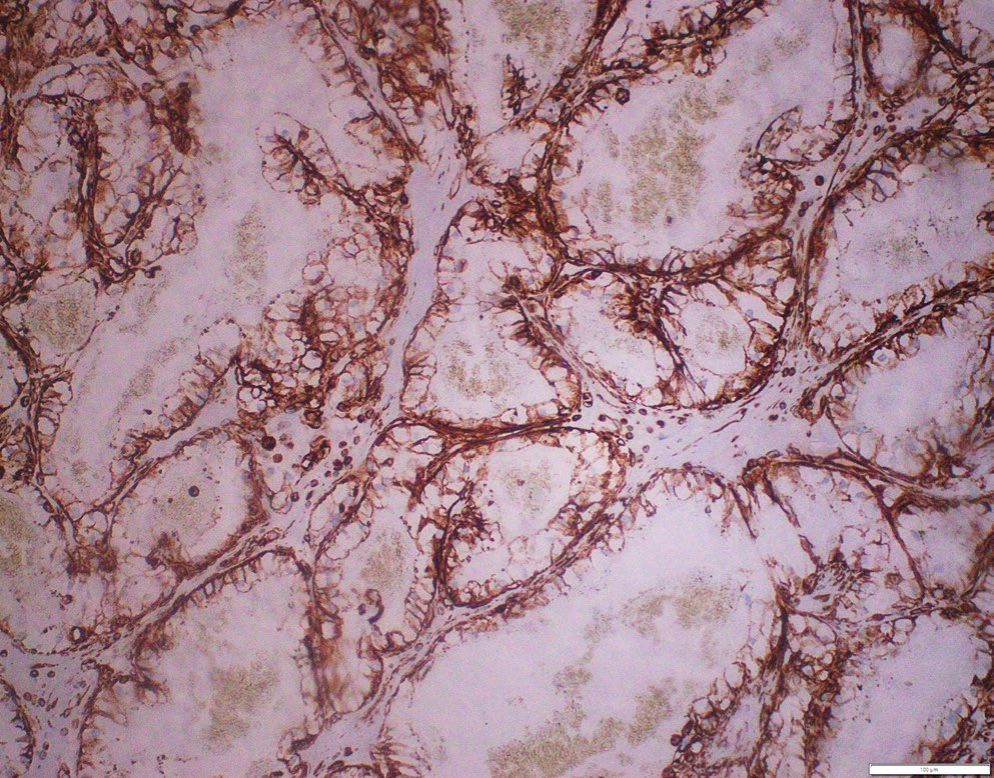
(200×): Vimentin: Immunoreactive in tumor cells

**Figure 5:**
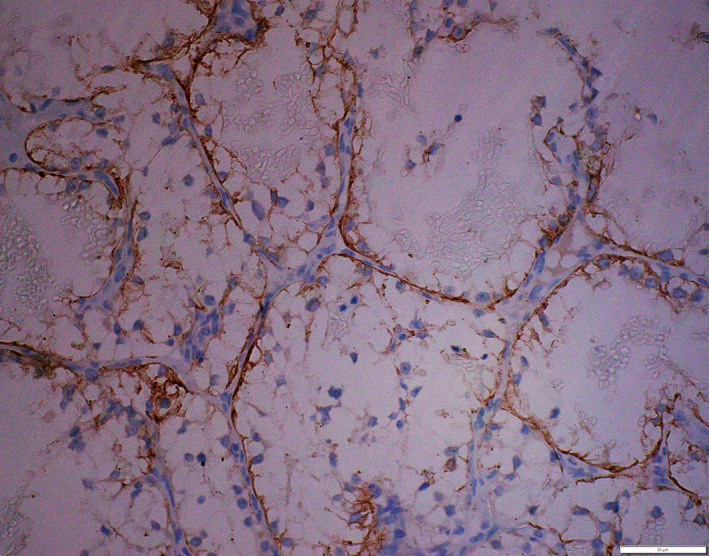
(400×): CD10: Immunoreactive in tumor cells

On immunohistochemistry, the tumor cells were immunoreactive for cytokeratin, CD10 and vimentin (Figures 4 and 5). Additional IHC markers like CD 34, factor VIII‐related antigen, S100, HMB45, and CEA were nonimmunoreactive. Vascular tumors were ruled out, as the tumor cells were immunoreactive for cytokeratin and negative for CD34 and factor VIII‐related antigen. Melanoma was ruled out, as the tumor cells were nonimmunoreactive for S100 protein and HMB45. Clear cell adenocarcinoma of various organs was ruled out as the tumor cell was immunoreactive to CD10 and nonimmunoreactive to CEA. Thus, a diagnosis of clear cell variant of renal cell carcinoma metastasis was established.

PET (positron emission tomography) scan revealed no residual disease at the primary site. Hypermetabolic right lower paratracheal and bilateral hilar lymphadenopathy—likely to be inflammatory, was noted. Fine needle aspiration cytology done from the same showed features of reactive lymphoid hyperplasia. There was no evidence of malignancy. The patient is under close follow‐up and is doing well and is disease free 6 months’ postsurgical resection.

## 3 DISCUSSION

RCC can show multiple distant site involvement via hematogenous spread. Overt metastasis is noted at the time of presentation in 20%‐30% of patients.^2^ RCC is also known for late recurrences; lesions can appear 10 years or more after initial surgical treatment.^3^

Skin metastases have been reported to occur in around 3% of renal tumors. Skin metastases of RCC are not easily identified because of the low suspicion index for these skin lesions, which usually mimic common dermatological disorders. They have been observed more commonly in males. Skin metastases mainly occur in the head, neck, and trunk.^4^ A total of 80%‐90% of patients with skin metastases are patients with a prior diagnosis of renal cell carcinoma. However, 10%‐20% of patients are diagnosed with skin lesions before the primary lesion is identified.^5^ Skin metastases are considered to have a poor prognosis, which is associated with synchronous visceral metastases in up to 90% of cases, resulting in tumor‐specific survival of usually shorter than six months.^6^ However, this case showed an isolated single site of metastasis, with no visceral involvement and thus a prolonged survival even after six months of resection of the metastatic lesion.

Lesions usually occur between 6 months and 5 years after the first diagnosis. Another distant metastases or recurrence of the tumor is found in the majority of patients. In our case, skin metastasis was detected 10 years after the first diagnosis, and no other metastatic focus or recurrence was detected. RCC skin metastasis is often a poor prognostic indicator, and the expected life span is less than six months as stated in few studies.^7^ However, the present case has survived disease free six months post skin metastasectomy.

Cutaneous clear cell RCC metastases are rare and are typically located in the head and neck region. Physical examination findings vary from small vascular‐appearing tumors to large ulcerative lesions.^8^ They are rapidly growing, round or oval‐shaped lesions, which can be of various colors ranging from normal skin color to a violaceous hue.^9^ Clinical presentation may be confused with hemangioma, basal cell carcinoma, melanoma, or pyogenic granuloma. There was a similar appearance of hemangioma in our case. The immunohistochemical examination provides a microscopic differential diagnosis.

Metastatic renal cell carcinoma therapy consists of surgical (radical nephrectomy) treatment, in selected patients and use of immune checkpoint inhibitors or anti‐angiogenic tyrosine kinase inhibitors (TKIs) depending on patients' risk. The treatment approach for single, isolated skin lesions is surgical removal of the lesion only. Radiotherapy may be an alternative to surgery in cases where surgical intervention is not feasible.^10^

## 4 CONCLUSION

Newly occurring skin lesions of renal cell carcinoma patients should be carefully evaluated. Although skin metastases are suggestive of a bad sign of progression, disease‐free follow‐up is possible after appropriate surgical excision.

## CONFLICT OF INTEREST

None declared**.**

## AUTHOR CONTRIBUTIONS

Dr Pretty Singh and Dr Kavita Somani: involved in diagnosing the case. Dr Pretty Singh: collected the data, wrote the article, and analyzed the results. Dr Kavita Somani: supported, analyzed and presented criticism to the paper.

## CONSENT FOR PUBLICATION

Written informed consent was obtained from the patient for publication of this case report and any accompanying images.
